# Oxidative stress induced by the anti-cancer agents, plumbagin, and atovaquone, inhibits ion transport through Na^+^/K^+^-ATPase

**DOI:** 10.1038/s41598-020-76342-5

**Published:** 2020-11-11

**Authors:** Yousef Alharbi, Arvinder Kapur, Mildred Felder, Lisa Barroilhet, Bikash R. Pattnaik, Manish S. Patankar

**Affiliations:** 1 Department of Obstetrics and Gynecology, University of Wisconsin-Madison, Madison, WI 53792 USA; 2 Department of Veterinary Medicine, Qassim University, Qassim, Saudi Arabia; 3 Department of Pediatrics, Ophthalmology and Visual Sciences, McPherson Eye Research Institute, University of Wisconsin-Madison, Madison, WI 53706 USA

**Keywords:** Cancer, Medical research, Oncology

## Abstract

Oxidative stress inhibits Na^+^/K^+^-ATPase (NKA), the ion channel that maintains membrane potential. Here, we investigate the role of oxidative stress-mediated by plumbagin and atovaquone in the inhibition of NKA activity. We confirm that plumbagin and atovaquone inhibit the proliferation of three human (OVCAR-3, SKOV-3, and TYKNu) and one mouse (ID8) ovarian cancer cell lines. The oxygen radical scavenger, N-acetylcysteine (NAC), attenuates the chemotoxicity of plumbagin and atovaquone. Whole-cell patch clamping demonstrates that plumbagin and atovaquone inhibit outward and the inward current flowing through NKA in SKOV-3 and OVCAR-3. Although both drugs decrease cellular ATP; providing exogenous ATP (5 mM) in the pipet solution used during patch clamping did not recover NKA activity in the plumbagin or atovaquone treated SKOV-3 and OVCAR-3 cells. However, pretreatment of the cells with NAC completely abrogated the NKA inhibitory activity of plumbagin and atovaquone. Exposure of the SKOV-3 cells to either drug significantly decreases the expression of NKA. We conclude that oxidative stress caused by plumbagin and atovaquone degrades NKA, resulting in the inability to maintain ion transport. Therefore, when evaluating compounds that induce oxidative stress, it is important to consider the contribution of NKA inhibition to their cytotoxic effects on tumor cells.

## Introduction

An uncontrolled increase in intracellular oxygen radicals can cause lipid, protein, and DNA damage leading to apoptotic cell death. Cancer cells are especially prone to damage that occurs due to the perturbation of cellular oxidative stress^1,2^. Therefore, agents that can increase intracellular oxygen radicals are being actively studied for the treatment of cancer.

Plumbagin is a naturally occurring naphthoquinone that inhibits the proliferation of prostate, pancreatic, ovarian, and endometrial cancers^3–10^. Previous reports indicated that plumbagin mediates its cancer cytotoxic activity by activating p53 and inhibiting NFκb, PKCε, and other important cell signaling mediators^11–13^. Recently, we demonstrated that the immediate effect of plumbagin on cancer cells is a rapid increase in the oxygen radical flux^13^. The reactive oxygen surge in plumbagin-treated cells causes double-strand breaks in the DNA and subsequent cell death by apoptosis^13^.

Our investigations into the mechanism of action of plumbagin indicate that this molecule, because of its naphthoquinone ring, can interfere with cellular redox reactions^13^. Plumbagin targets oxidative phosphorylation- the primary generator of oxygen radicals. The similarities in the structure of plumbagin and ubiquinone likely allow this molecule to interfere with the transfer of electrons to molecular oxygen and, as a result, generate an intracellular oxygen radical flux that contributes to cell death^13^. With the cancer cells already experiencing higher levels of oxidative stress as compared to normal cells due to increased metabolism, the increase in intracellular oxygen radicals in response to plumbagin results in overwhelming oxidative damage and may explain the potent anti-cancer effects of this natural product.

Plumbagin treatment results in increased expression of p53 and phosphorylation of its Serine-15 residue^11^. The p53-regulated genes, PUMA and NOXA, are also upregulated following plumbagin treatment. Inhibition of oxidative damage by N-acetylcysteine (NAC) prevents p53 activation in plumbagin-treated cells^13^. Furthermore, inhibition of p53 by its specific inhibitor, pifithrin-α, also blocks the cytolytic activity of plumbagin^13^. These experiments indicate that the rapid increase in intracellular oxygen radical flux is a central cellular insult that activates the apoptotic effects of p53.

On the other hand, plumbagin can induce apoptosis in cancer cells that lack p53 expression^13^. These studies suggest that p53 activation is not obligatory for plumbagin-mediated cell death. However, even in the p53-negative cells, neutralization of the oxygen radicals by NAC blocks plumbagin-mediated cytotoxicity^13^. The oxygen radicals produce pleiotropic effects. Therefore, the apoptotic activity of plumbagin cannot be ascribed to one specific pathway but rather is the net result of the various cell death pathways activated by the intracellular oxygen radicals.

The Na^+^/K^+^-ATPase (NKA) is a ubiquitous ion transport channel that is necessary for the maintenance of membrane potential^14^. Several studies have indicated that the NKA is sensitive to oxidative stress. For example, decreased expression of NKA has been reported in cells undergoing oxidative damage due to hypoxia^15–20^. Recently, we demonstrated that plumbagin inhibits NKA activity in canine cancer cells^21^. Based on these studies, we suggested that plumbagin’s ability to induce a rapid increase in the level of intracellular oxygen radicals, compromised the canine cancer cells to maintain Na^+^/K^+^ ionic balance. Lack of regulation of NKA may also contribute to cell death upon treatment with plumbagin. Our focus on canine cancer cells was to support the testing of plumbagin and other oxidative stress causing drugs in preclinical dog models. The current study was initiated to demonstrate that the oxidative stress initiated by plumbagin also inhibits NKA activity in human cancer cell lines. It is critical to demonstrate that plumbagin has similar effects on NKA in canine and human cell lines to support preclinical studies in dog models.

We further show that this mechanism of NKA inhibition in human cancer cells is not restricted to plumbagin, but is shared by atovaquone, an FDA-approved anti-malarial agent, which also causes oxidative damage and is an excellent candidate for repurposing as a chemotherapeutic drug^22,23^.

## Results

### Plumbagin inhibits the proliferation of human and mouse ovarian cancer cell lines

**Figure 1:**
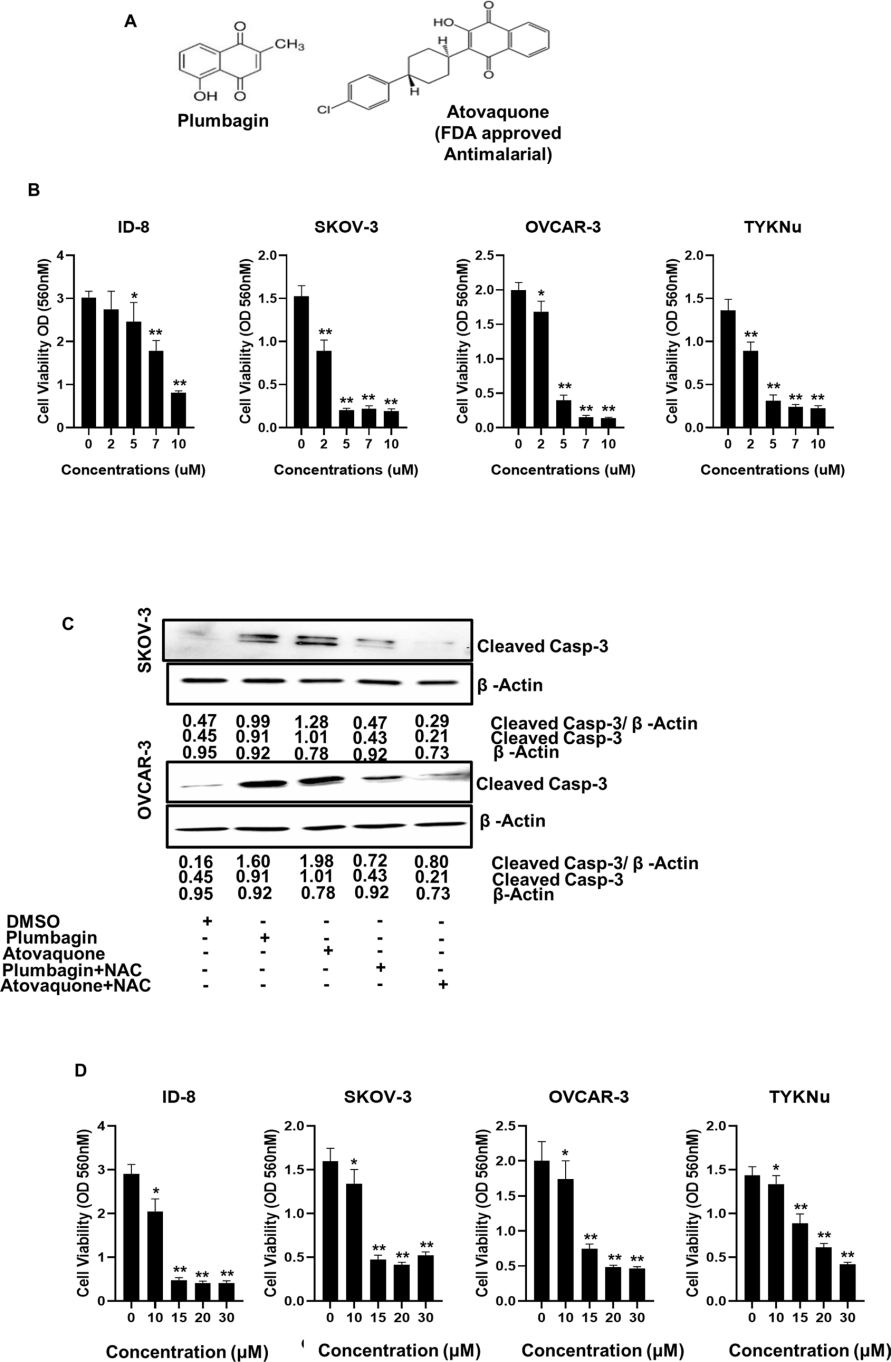
Plumbagin and atovaquone inhibit proliferation of ovarian cancer cells. (**A**) The chemical structures of plumbagin and atovaquone are shown. Both molecules have the naphthoquinone substructure that mimics ubiquinone. (**B**) Effect of plumbagin on the viability of four ovarian cancer cell lines as determined by MTT assays is shown. Each bar is an average of three biological replicates. (**C**) Western blot analysis of SKOV-3 and OVCAR-3 cells treated with plumbagin (10 μM) and atovaquone (10 μM) for 48 h. As shown in the legend below the blots, in some experiments the cell lines were pre-loaded with NAC (2 mM) for 30 min before treatment with plumbagin or atovaquone or incubation with media that contained only the vehicle (DMSO). The numerical values show the density of the cleaved caspase 3 or the β-actin bands. The ratio of densitometry for cleaved caspase 3 to β-actin is also shown. Each blot is representative of three biological replicates. (**D**) MTT assay results showing the inhibitory effect of atovaquone on the viability of the four ovarian cancer cell lines. Each bar is an average of three biological replicates. *p = 0.012 and **p = 0.0002.

Recently we demonstrated that plumbagin (Fig. 1A) was an effective anti-cancer agent and that its cytotoxic effect could be demonstrated across multiple cancer cell lines. Here, we extend these previous observations to further confirm the cytolytic activity of plumbagin against mouse (ID8) and human (SKOV-3, OVCAR-3, and TYKNu) high grade serous ovarian cancer cell lines. Our data demonstrate that irrespective of the cell line used, plumbagin was able to inhibit proliferation at an IC_50_ between 2.5 and 10 µM. (Fig. 1B).

Exposure to plumbagin results in a rapid increase in intracellular oxygen radicals^13^. The oxidative stress caused by the oxygen radical flux triggers cell death. We confirmed this observation by demonstrating that pretreatment of OVCAR-3 and SKOV-3 cells with the oxygen radical scavenger, NAC, inhibited plumbagin-mediated apoptosis (Fig. 1C).

Atovaquone is an FDA approved drug that is widely used to prevent malaria infections. In the Plasmodium parasite, atovaquone inhibits electron transport at the level of mitochondrial complex III. In our on-going studies, we have also determined that the anti-malarial drug, atovaquone (Fig. 1A), uses a mechanism that is similar to plumbagin and causes cancer cell death via the generation of intracellular oxygen radicals (Kapur et al. manuscript in preparation). Here, we provide evidence that atovaquone inhibits proliferation of ID8, SKOV-3, OVCAR-3, and TYKNu cells (Fig. 1D). Additionally, we also show that neutralization of the oxygen radical surge by NAC blocks apoptosis in atovaquone-treated cells (Fig. 1C).

### Effect of plumbagin and atovaquone on NKA activity

**Figure 2:**
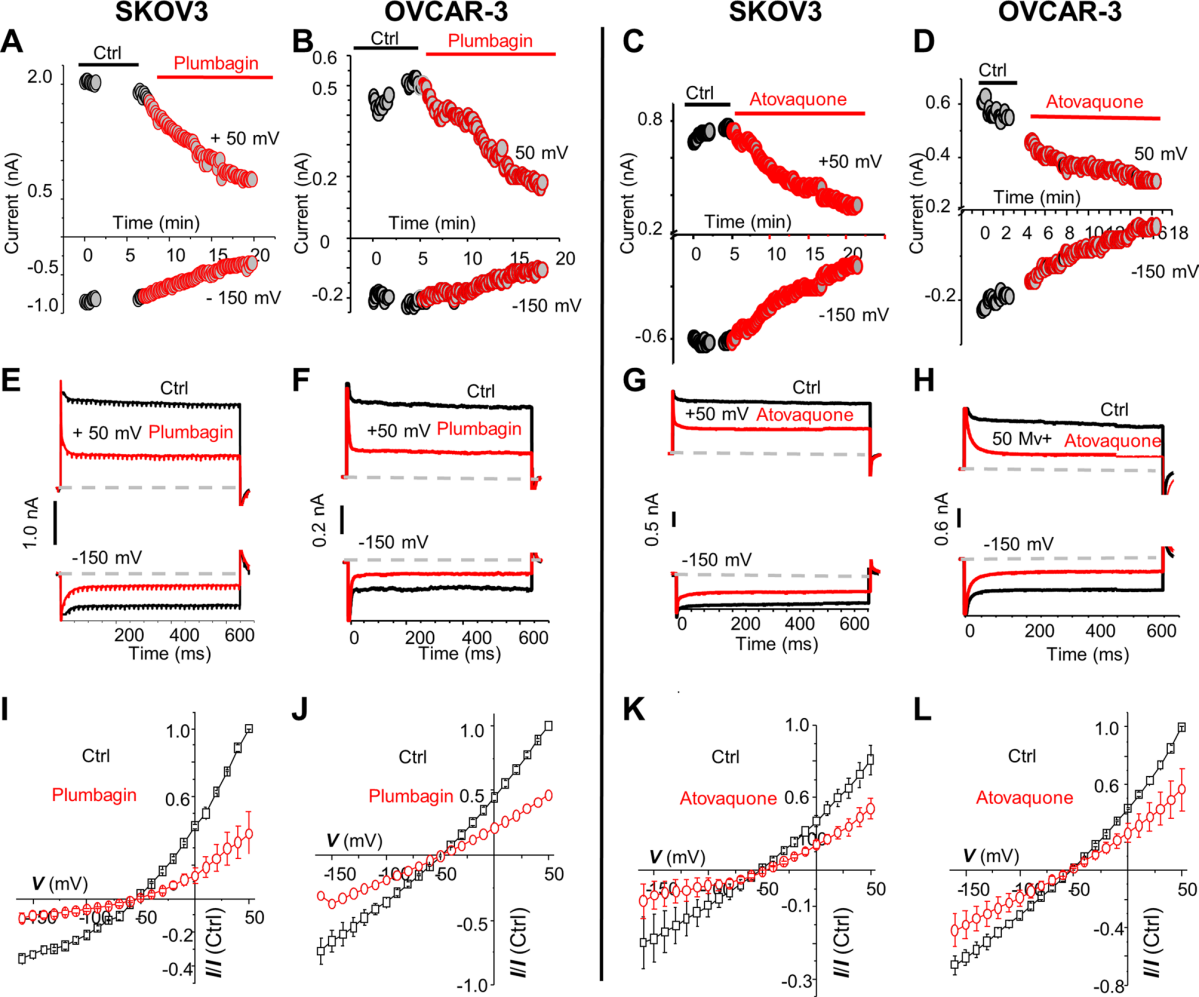
Plumbagin and atovaquone inhibit ionic current through NKA. SKOV-3 and OVCAR-3 cells used for the patch-clamp experiment were treated with 10 µM plumbagin (**A**, **B**, **E**, **F**, **I** and **J**) or 10 µM atovaquone (**C**, **D**, **G**, **H**, **K**, and **L**). Na^+^/K^+^ ion current was measured in the treated and control cells. (**A–D**) Time course measurement of NKA ion current over a 20 min duration. The black circles is for cells before treatment, and red circles shows current measurements after the cells were treated with plumbagin or atovaquone. The outward and inward currents were measured at + 50 mV and − 150 mV, respectively. Data shown is for one representative cell. All experiments were repeated on 4–6 cells. (**E–H**) Representative current traces from a representative SKOV-3 or OVCAR-3 cell treated with plumbagin or atovaquone are shown (red trace). The black trace represents the control recording from untreated SKOV-3 or OVCAR-3 cells. The voltage pulse steps were applied in 21 steps from + 50 to − 150 mV starting from the holding potential of − 60 mV. The vertical scale bar shows the current in nA. (**I–L**) The average of normalized current–voltage (I–V) curve in control (black trace) and after the application of plumbagin or atovaquone (red trace) are shown. The results indicate a 53.1% and 46% inhibition of current in SKOV-3 (n = 4 cells; p-value = 0.002057) and OVCAR-3 (n = 6; p value = 0.0145) cells treated with plumbagin, respectively. For atovaquone treatment the inhibition of current in SKOV-3 and OVCAR-3 was 56.1% (n = 4; p-value = 0.0077) and 39% (n = 4; p-value = 0.000721), respectively.

To determine the effect of plumbagin and atovaquone on ion transport through NKA, we performed electrophysiology experiments on two established human cancer cell models, SKOV-3 and OVCAR-3. Whole-cell patch-clamp was performed on single cultured cells in the presence of pipette and bath solutions that isolated NKA current. Average current–voltage plots from 4 to 6 cells were plotted and analyzed to monitor the NKA inhibitory activity of plumbagin and atovaquone (Fig. 2).

Treatment of SKOV3 and OVCAR-3 cells with plumbagin (10 µM) or atovaquone (10 μM) causes a slow decrease in both inward and outward current (Fig. 2A–D). For example, the outward current at + 50 mV in the untreated SKOV-3 and OVCAR-3 cells was 1.98 nA and 0.84 nA, respectively (Fig. 2A,B, black circles). Upon plumbagin treatment (Fig. 2A,B, red circles), the measured current decreased to 0.7 nA and 0.5 nA, for SKOV-3 and OVCAR-3 cells, respectively. Similarly, the inward current at − 150 mV in the SKOV-3 cells before and after plumbagin treatment was − 0.8 nA and −0.3 nA, respectively, and in OVCAR-3 cells the control and test readings were − 0.5 nA and − 0.34 nA, respectively (Fig. 2A,B). Inhibition of NKA activity was also observed in atovaquone-treated SKOV3 (0.7 nA versus 0.34 nA in the control) and OVCAR-3 (0.6 nA versus 0.3 nA in the control) cells at + 50 mV (Fig. 2C,D).

To determine if the current was time or voltage-dependent, we conducted the electrophysiology experiments using a discontinuous voltage gradient. As shown in Fig. 2E–H, neither outward nor inward current was time or voltage-dependent at − 150 to + 50 mV in plumbagin or atovaquone treated cells. The measured current at + 50 mV in the control SKOV-3 and OVCAR-3 cells from the plumbagin experiment was 1.9 nA and 0.8 nA, respectively (Fig. 2E,F). When treated with plumbagin, the current decreased to 0.7 nA and 0.4 nA in SKOV-3 and OVCAR-3, respectively (Fig. 2E,F, red trace). When the voltage was maintained constant at − 150 mV, the current measured in SKOV-3 and OVCAR-3 was − 0.8 nA and − 0.5 nA, respectively (Fig. 2E,F, black trace). In comparison, plumbagin treatment decreased the inward current measured at a constant voltage of − 150 mV to − 0.3 and − 0.34 nA in SKOV-3 and OVCAR-3, respectively (Fig. 2E,F red trace). A similar level of inhibition of NKA was also observed in SKOV-3 and OVCAR-3 cells treated with atovaquone (Fig. 2G,H). The measured current at + 50 mV in the control SKOV-3 and OVCAR-3 cells were 0.69 nA and 0.60 nA, respectively. After atovaquone treatment, the currents decreased to 0.34 nA and 0.30 nA, respectively (Fig. 2G,H). At − 150 mV, the control current in SKOV-3 and OVCAR-3 were − 0.59 nA and − 0.43 nA, and upon atovaquone treatment, the current decreased to 0.31 nA and − 0.23 nA respectively (Fig. 2G,H). These results indicated that plumbagin and atovaquone inhibit NKA-mediated ion transport. Average current–voltage plots from 4 to 6 recorded cells demonstrate the overall inhibitory activity (> 50% inhibition in all experiments) of plumbagin and atovaquone on NKA (Fig. 2 I–L).

### Inhibition of NKA by plumbagin and atovaquone is not due to a decrease in intracellular ATP

In our on-going work, we observed that plumbagin and atovaquone inhibit oxidative phosphorylation, and as a result, cause a significant decrease in intracellular ATP levels in the cancer cells^13^. We, therefore, reasoned that the decrease in inward and outward current observed in plumbagin and atovaquone-treated SKOV3 and OVCAR-3 could be a result of decreased availability of ATP leading to suboptimal NKA ion channel activity. To directly address this potential mechanism, we conducted electrophysiology experiments where the pipet solution was supplemented with 5 mM ATP. This ensured that there was sufficient intracellular ATP for optimal NKA activity.

**Figure 3:**
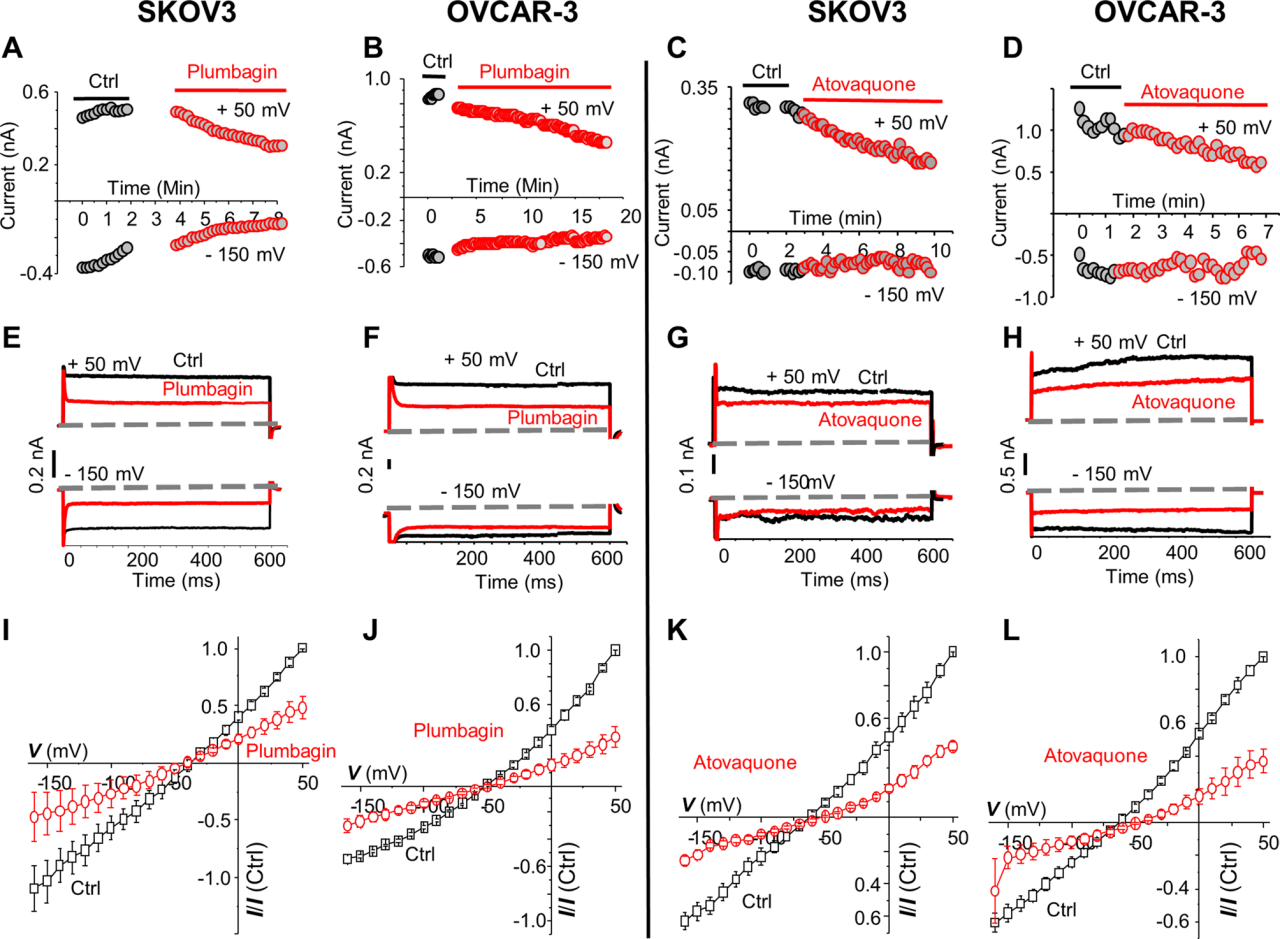
ATP supplementation does not prevent NKA inhibition by plumbagin or atovaquone. The glass micropipette used for patch clamping was supplemented with (5 mM ATP). Na^+^/K^+^ current through the NKA was measured in SKOV-3 (**A**, **C**, **E**, **G**, **I** and **K**) and OVCAR-3 (**B**, **D**, **F**, **H**, **J**, and **L**) cells with and without treatment with plumbagin or atovaquone (10 µM). (**A–D**) Time course during 20 min shows the measurement of NKA ion currents in SKOV-3 and OVCAR-3 for all of the control and treatment conditions. The constant outward and inward currents were measured at 50 mV and − 150 mV, respectively. Representative plots for one out of 4–6 cells are shown. (**E–H**) Representative current trace from one ATP supplemented SKOV-3 or OVCAR-3 cell treated with plumbagin or atovaquone are shown (red trace). The black trace represents the control recording for SKOV-3 and OVCAR-3 cells that were supplemented with ATP but were not treated with plumbagin or atovaquone. The voltage pulse steps were applied in 21 steps from 50 to − 150 mV starting from the holding potential of − 60 mV. The vertical scale bar shows the current in nA. (**I–L**) The average of normalized current–voltage (I–V) curve in ATP supplemented cells in bath solution (black trace) or upon treatment with plumbagin or atovaquone (red trace) are shown. The results indicate a 53.1% and 50.22% inhibition of current in SKOV-3 (n = 5 cells; p-value = 0.00018) and OVCAR-3 (n = 6; p-value = 0.0038) cells treated with plumbagin, respectively. For atovaquone treatment the inhibition of current in SKOV-3 and OVCAR-3 was 56.8% (n = 4; p-value = 0.000198) and 63% (n = 5; p-value = 0.000175), respectively.

However, even upon ATP supplementation, plumbagin and atovaquone continued to inhibit the inward and outward currents in SKOV3 and OVCAR-3 cells (Fig. 3). The outward current at + 50 mV in the untreated SKOV-3 and OVCAR-3 cells were 0.46 and 0.84 nA, respectively (Fig. 3A,B, black trace). Plumbagin treatment of SKOV-3 and OVCAR-3 decreased current amplitude to 0.25 nA and 0.46 nA, respectively (Fig. 3A, B, red circles). A similar pattern of inhibition was observed when both cell types were exposed to atovaquone even when the cells were supplemented with exogenous ATP (Fig. 3C, D).

At + 50 mV, the current measured in untreated SKOV-3 and OVCAR-3 cells was 0.3 nA and 1.1 nA, respectively, and the current decreased to 0.17 nA and 0.6 nA upon exposure to atovaquone, respectively (Fig. 3C,D). This inhibition of NKA activity by plumbagin and atovaquone in the presence of ATP supplementation was also observed when the voltage was applied in a stepwise gradient (Fig. 3E–H). The average of 5–6 cells plotted in I–V plots showed that both drugs continued to inhibit NKA activity (> 50% inhibition in all experiments) even in the presence of ATP in the pipet solution (Fig. 3 I–L).

### Oxygen radical scavenger, NAC abrogates NKA inhibitory activity

Plumbagin and atovaquone interfere with ubiquinone’s ability to transfer electrons in the oxidative phosphorylation pathways. As a result, there is an insufficient transfer of electrons to molecular oxygen resulting in a rapid intracellular oxygen radical flux. Previous reports have indicated that the NKA complex is sensitive to cellular oxidative stress (caused by an unopposed upregulation of intracellular reactive radicals). Oxidative modification and subsequent proteolytic degradation of the NKA results in an inability of the cells to maintain membrane potential. We, therefore, tested if the reduction of the intracellular oxygen radical flux in plumbagin and atovaquone-treated cells could attenuate NKA inhibition mediated by these two drugs.

**Figure 4:**
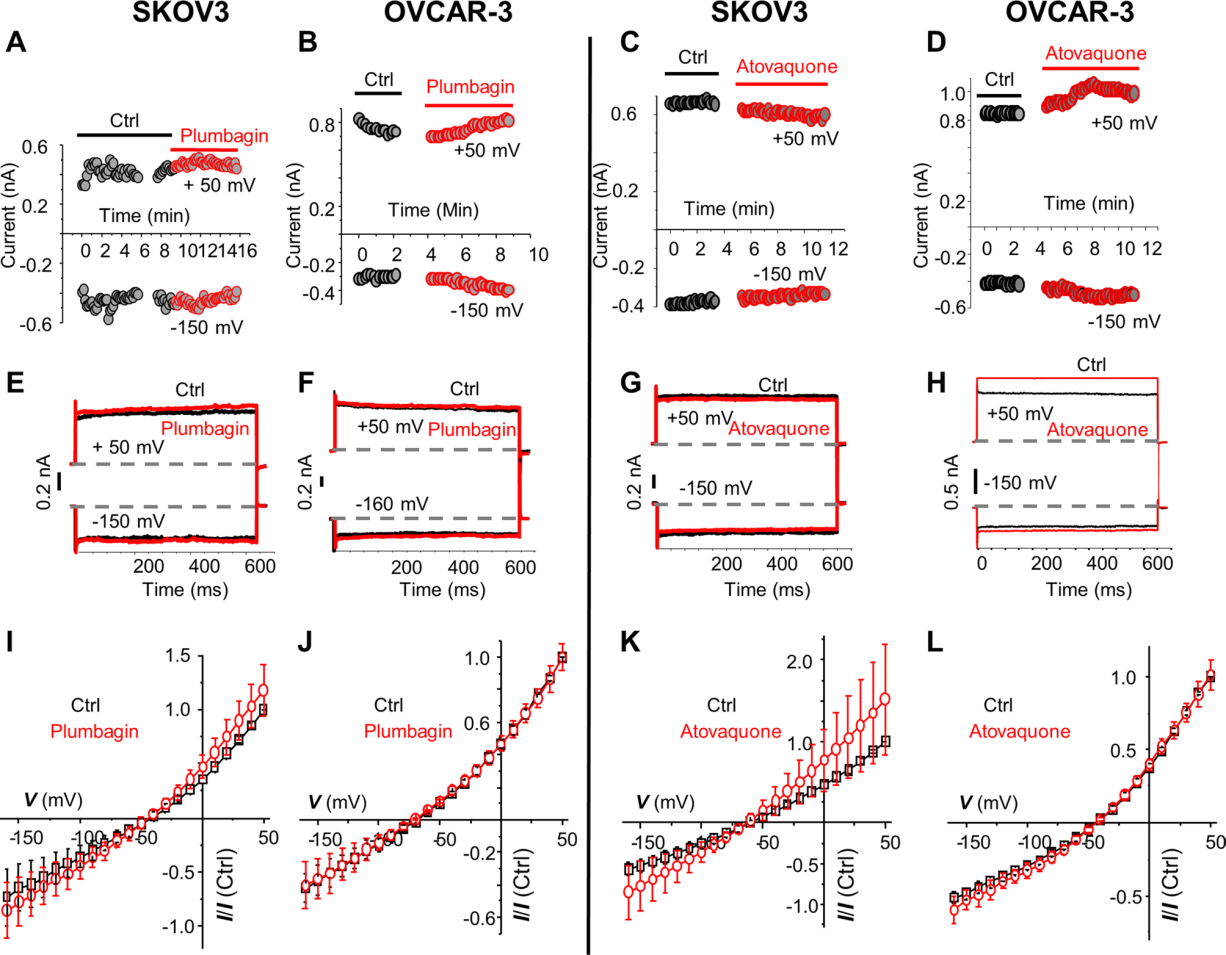
Oxidative stress-induced by plumbagin and atovaquone inhibits NKA activity. SKOV-3 (**A**, **C**, **E**, **G**, **I** and **K**) and OVCAR-3 (**B**, **D**, **F**, **H**, **J**, and **L**) cells were preincubated with 2 mM NAC for 1 h. Healthy cells were patch clamped before and after treatment with plumbagin or atovaquone (10 μM). (**A–D**) Time course during 20 min shows the measurement of NKA ion currents in NAC preincubated SKOV-3 and OVCAR-3 for all of the control (black circles) and treatment (red circles) conditions. The constant outward and inward currents were measured at + 50 mV and − 150 mV, respectively. (**E–H**) Representative current trace from one NAC preincubated SKOV-3 or OVCAR-3 cell treated with plumbagin or atovaquone are shown (red trace). The black trace represents the control recording for SKOV-3 and OVCAR-3 cell that was preincubated with NAC but not treated with plumbagin or atovaquone. The voltage pulse steps were applied in 21 steps from + 50 to − 150 mV starting from the holding potential of − 60 mV. The vertical scale bar shows the current in nA. (**I–L**) The average of normalized current–voltage (I–V) curve in NAC preincubated cells in bath solution (black trace) or upon treatment with plumbagin or atovaquone (red trace) are shown. The results indicate no statistically significant difference in the current in SKOV-3 (n = 5; p-value = 0.509) and OVCAR-3 (n = 4; p-value = 0.995) cells treated with plumbagin. Similarly, no inhibition of NKA current was observed in the SKOV-3 (n = 5; p-value = 0.6) and OVCAR-3 (n = 4; p-value = 0.937) cells treated with atovaquone.

SKOV3 and OVCAR-3 cells were pre-loaded for 1 h with the oxygen radical scavenger, NAC. After washing away the excess NAC, NKA current in the cells was recorded while treated with vehicle (control), plumbagin (10 µM), or atovaquone (10 µM). In both cell lines, the pretreatment with NAC resulted in an almost complete loss of NKA inhibitory activity of plumbagin and atovaquone (Fig. 4). For example, in NAC-loaded SKOV3 cells, the outward current at + 50 mV in the controls was 0.33 nA, and upon treatment with plumbagin, the current measured 0.44 nA (Fig. 4A). Similarly, the outward current in atovaquone treated SKOV3 cells measured 0.82 nA, which was not significantly different than that measured in the corresponding untreated SKOV3 cells (0.83 nA) (Fig. 4C). Recording of OVCAR-3 cells also demonstrated that NAC pretreatment reversed the inhibition of the outward current by plumbagin (0.62 nA versus 0.7 nA in untreated control) and atovaquone (1.0 nA versus 0.85 nA in untreated control) (Fig. 4A–D). Voltage gradient and the average current recordings of these experiments confirmed that pretreatment with NAC reversed NKA inhibition by plumbagin and atovaquone in SKOV-3 and OVCAR-3 cells (Fig. 4E–L).

**Figure 5:**
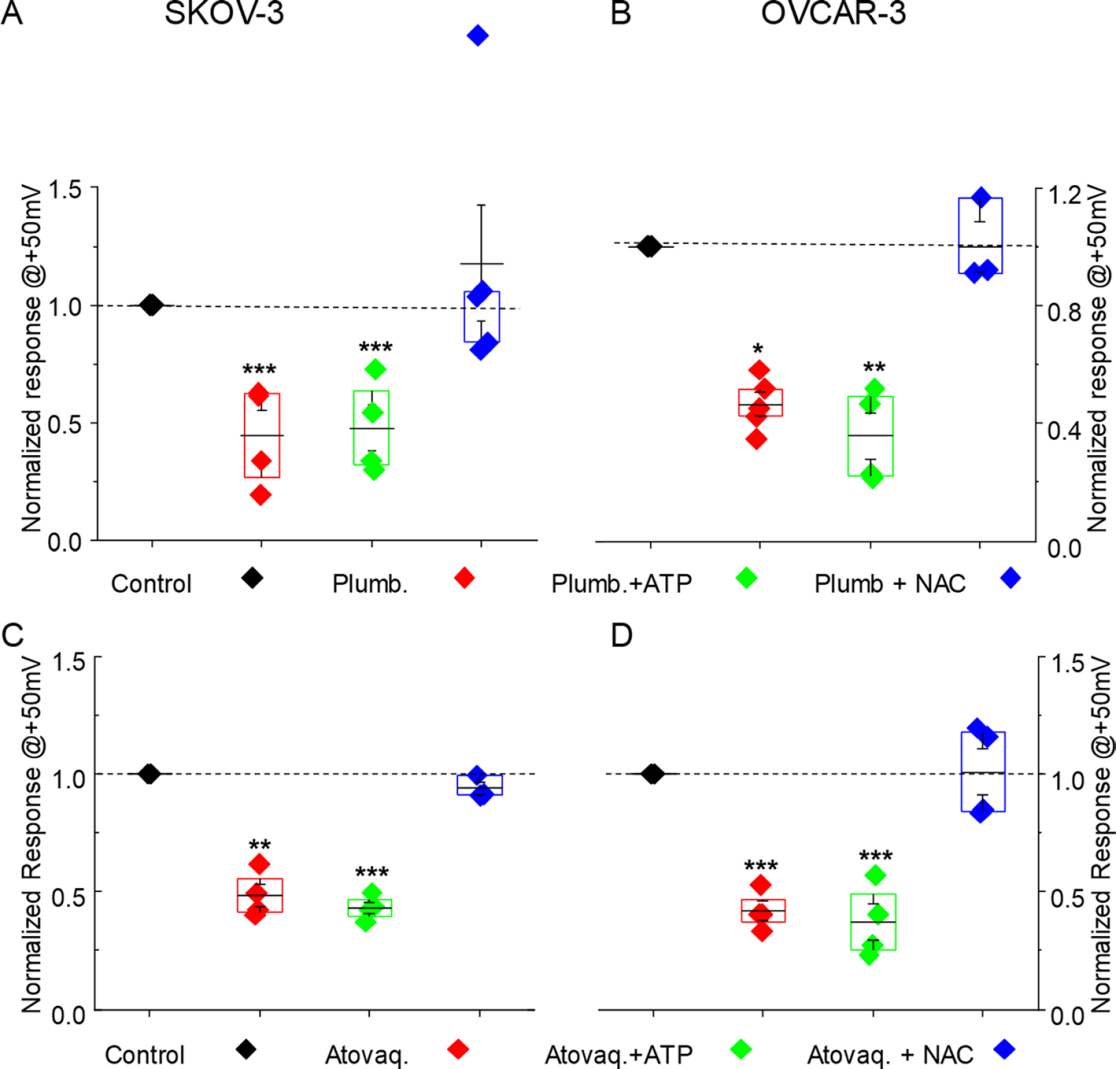
Normalized current at + 50 mV in response to plumbagin and atovaquone. The average outward current measured at + 50 mV for all of the SKOV-3 (**A**,**C**) and OVCAR-3 (**B**,**D**) cells monitored in our electrophysiology experiments is shown. Effect of plumbagin (**A**,**B**) and atovaquone (**C**,**D**) as single agents (red diamonds) or in cells simultaneously treated with ATP (green diamonds) or NAC (blue diamonds) is shown as normalized current. The black diamonds show current values for untreated SKOV-3 and OVCAR-3 cells maintained in the bath solution. SKOV-3 (plumbagin *p = 0.002057 , plumbagin + ATP **p = 0.00018, atovaquone, ^#^p = 0.0077, Atovaquone + ATP, ^##^p = 0.000198). OVCAR-3 (plumbagin ***p = 0.0145, plumbagin + ATP ****p = 0.0083, Atovaquone ^###^p = 0.000721, Atovaquone + ATP ^####^p = 0.000175). ns, not significant.

The average response to plumbagin and atovaquone from all of the electrophysiology experiments is summarized in Fig. 5 as normalized current at + 50 mV. In SKOV-3 (Fig. 5A,C) and OVCAR-3 (Fig. 5B,D), plumbagin or atovaquone inhibited NKA current by 40–50%. Supplementation of cell cytoplasmic milieu with ATP did not prevent the inhibitory effect of plumbagin or atovaquone. Upon neutralization of cellular oxidative stress with NAC, NKA inhibition by plumbagin and atovaquone was reversed.

### Plumbagin and atovaquone treatment decrease the expression of NKA

**Figure 6:**
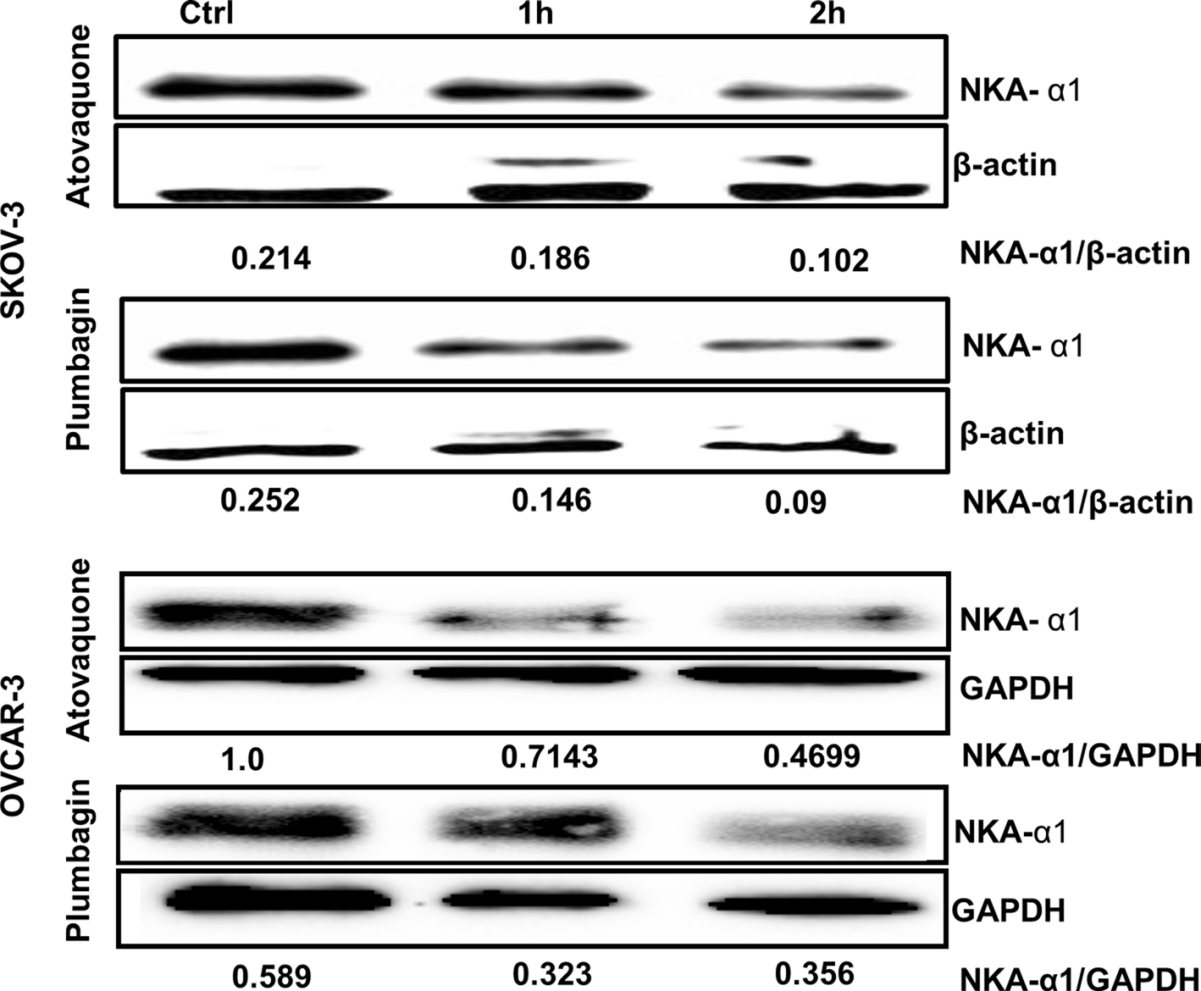
Plumbagin and atovaquone treatment decreases NKA expression. SKOV-3 and OVCAR-3 cells were treated with plumbagin (10 μM) or atovaquone (10 μM) for 1 and 2 h. The expression of NKA-α1 subunit at these timepoints was determined by western blotting. The numerical values below the blot show densities of the bands for NKA or the loading controls, β-actin or GAPDH. The densitometry for ratios of NKA-α1 and β-actin or GAPDH are also provided. The blot is a representative of two independent replicates for each cell line.

Under hypoxic conditions, NKA expression is decreased, resulting in loss of membrane potential. We, therefore, tested if treatment of the cancer cells with plumbagin and atovaquone was triggering downregulation of NKA expression. We demonstrate that in SKOV3 and OVCAR-3 cells, plumbagin and atovaquone decreased the expression of the α1 subunit of NKA at relatively short time points (1 and 2 h) (Fig. 6). This decrease in NKA-α1 expression correlates with the inhibition of NKA ion transport activity observed in our whole-cell patch-clamp experiments (Figs. 2, 3, 4, 5).

## Discussion

In this study, we conclusively demonstrate that the naturally occurring naphthoquinone, plumbagin, inhibits NKA in human cell lines and, therefore, negatively affects the ability of the cancer cells to maintain their membrane potential. Additionally, our results also indicate that the FDA-approved naphthoquinone, atovaquone, also inhibits ion transport through NKA. This is the first study to demonstrate the inhibitory effect of atovaquone on NKA. Our studies demonstrate the mechanism by which plumbagin and atovaquone can inhibit NKA. This ion transporter plays an essential role in maintaining membrane potential^14^. The best small-molecule agents that can inhibit NKA activity are the cardiac glycosides that are composed of a sterol moiety linked to a glucoside^14,24^. Plumbagin and atovaquone do not share structural similarities with the known cardiac glycoside inhibitors of NKA^23–27^. Therefore, it is unlikely that plumbagin and atovaquone can serve as direct inhibitors of NKA. Instead, our results indicate that the oxidative stress induced by plumbagin and atovaquone is responsible for inhibiting the ion transport activity of NKA.

Plumbagin and atovaquone, because of their naphthoquinone unit, can mimic ubiquinone- both structurally as well as in terms of their chemical reactivities^13,23^. Atovaquone is an FDA-approved antimalarial agent that inhibits electron transport in the mitochondrial complex III of the electron transport chain^13,28^. Emerging data demonstrate that atovaquone inhibits electron transport not only in the malarial parasite but also in tumor cells^28^.

In a previous study, we demonstrated that plumbagin, similar to atovaquone, also inhibits electron transport in the mitochondria^13^. Immediately after cancer cells are exposed to plumbagin, there is a significant increase in the intracellular oxygen radicals^13^. We have demonstrated that the ensuing oxidative stress triggers apoptotic cell death pathways^13^. Plumbagin treatment decreases the oxygen consumption rate of cells and also decreases the intracellular NADH and ATP levels^13^. These experiments indicate that plumbagin inhibits oxidative phosphorylation in cancer cells. We propose investigating, plumbagin and its analogs for the treatment of ovarian and other solid tumors. Our on-going research on atovaquone also demonstrates an increase in intracellular oxygen radical flux and a decrease in cellular NADH and ATP when cancer cells are exposed to this drug (Kapur et al. manuscript in preparation). These effects of plumbagin and atovaquone are rapid as they occur within minutes of exposure.

NKA transports sodium ions to the extracellular environment and potassium ions to the cytoplasm. Transfer of both these ions is against the concentration gradient, and therefore NKA requires energy (ATP) to facilitate this transport. The ability of plumbagin and atovaquone to decrease cellular ATP levels subsequently reduces the amount of available energy to drive ion transport via NKA. However, when cells were supplemented with cytoplasmic ATP, both plumbagin and atovaquone continued to inhibit ion transport via NKA (Figs. 3 and 5). These results indicate that the lack of sufficient cellular energy was not the cause for the inhibition of NKA in the plumbagin and atovaquone-treated cancer cells.

Instead, the NKA inhibitory activity of plumbagin and atovaquone was completely abrogated when the cells were supplemented with the oxygen radical scavenger, NAC (Figs. 4 and 5). These results indicate that the oxidative stress induced by plumbagin and atovaquone is the most likely reason for the inhibition of NKA by these two agents.

Additionally, our experiments with NAC also demonstrate that plumbagin and atovaquone are not inhibiting the NKA like the cardiac glycosides that block ion transport by interacting with the ion-binding site of the complexes^29,30^. If this were the case, pretreatment of the cells with NAC would not be able to reverse the inhibition of NKA. Additionally, we also demonstrate that plumbagin and atovaquone cause downregulation of NKA expression on the cancer cells (Fig. 6), further evidence that the mechanism of these two drugs is distinct from that of the cardiac glycosides.

There are several reports demonstrating the harmful effects of oxygen radicals on NKA. Reactive oxygen species are known to inhibit the activity of the NKA in not just the cancer cells but in many different cell types, and antioxidant enzymes or inhibitors of the oxidative stress can attenuate reactive oxygen radical-mediated inhibition of NKA activity^15–20^. For example, in RPT cells, treatment with cadmium chloride (5 µM) leads to a rapid rise in intracellular oxygen radicals. These radicals were demonstrated to inhibit NKA^31^.

The cysteine and tyrosine residues located in the alpha subunit of NKA are sensitive to oxidative damage^32^. Oxidation of these residues can lead to degradation of the NKA complex. Oxygen radicals also are known to activate PKCζ, a kinase that can phosphorylate the α1 subunit of NKA^33^. This phosphorylation of the complex results in its endocytosis and degradation. Our studies indicate that the perturbation of membrane potential through the inhibition of NKA activity contributes to the chemotoxicity of plumbagin, atovaquone, and their analogs that trigger oxidative stress by interfering with electron transport in the oxidative phosphorylation pathway. These results can be used to develop novel drugs for the treatment of solid tumors.

## Materials and methods

### Cell culture, antibodies, and cell authentication

Ovarian cancer cells, SKOV-3 and OVCAR-3, were obtained from ATCC (SKOV-3 and OVCAR-3), and the TYKNu cells were a gift from Dr. Hillary Kenny, University of Chicago, IL. The ID8 cells were from Dr. Katherine Roby (University of Kansas, KS). SKOV3 and OVCAR-3 cells were maintained in RPMI-1640 media supplemented with 10% fetal calf serum and other chemicals and reagents as per the recommendation of ATCC. TYKNu and ID8 were maintained in DMEM media supplemented with 10% fetal calf serum. All cells were grown at 37 °C in a 5% CO_2_ environment. The cells were checked for mycoplasma every three months, and cells from passage numbers less than fifteen were used in all experiments. Authentication of the cells was done through STR analysis conducted by the Cytogenetics core of our institution. Cell line authentication was conducted no more than six months before their use in experiments.

Primary antibodies against pro- and cleaved caspase 3 and β-actin were purchased from Cell Signaling Technologies (Danvers, MA, USA) and horseradish peroxidase-conjugated sheep anti-mouse and goat anti-rabbit secondary antibodies were purchased from Jackson ImmunoResearch (West Grove, PA, USA). All other reagents were from ThermoFisher (Waltham, MA, USA).

### Cell viability assay

The sensitivity of cancer cell lines to plumbagin and atovaquone was assessed by MTT colorimetric assay. Cells (1 × 10^4^/well) were seeded in a flat-bottomed 96-well plate and incubated overnight in a humidified incubator at 37 °C and in 5% CO_2_ environment. The cells were then treated with various concentrations of plumbagin or atovaquone for 72 h. Control cells were treated with the vehicle, DMSO, for the matching time point. Following incubation with the drugs or the vehicle, MTT reagent (50 μg/ml final concentration/well) was added to each well, and the plates were incubated at 37 °C for 3 h. Subsequently, 100 μl of DMSO was added to each well to dissolve the formazan crystal, plates were mixed in a horizontal shaker for 2 min. The optical densities (OD) of the wells was measured at 560 nm on a microplate reader.

### Western blot

The SKOV-3 and OVCAR-3 cells (5–10 × 10^6^) were plated in a 10 cm diameter tissue culture plates. The cells were treated with plumbagin, atovaquone, or vehicle (DMSO) for short (1–2 h) and long (48 h) time points. Following treatment, the cells were lysed with RIPA (25 mM Tris-HCl pH 7.4 containing 150 mM sodium chloride, 0.1% sodium dodecyl sulfate, 0.5% sodium deoxycholate and 15 Triton X-100) containing protease inhibitor cocktail (10X protease inhibitors in the stock solution). The lysates were harvested from the dish, sonicated for 30 s and centrifuged for 30 min at 4 °C. The lysates were frozen until further use. Before conducting Western blotting, the protein concentration of each lysate was determined using a micro BCA assay (ThermoFisher, IL, USA). The lysate (30 μg/lane) was typically loaded in each lane of an SDS-PAGE gel. Depending on the molecular weight of the targeted protein, either 7.5 or 12% polyacrylamide gels were run at constant voltage (150 mV) for 1 h in running buffer (25 mM Tris, 119 mM Glycine and 1 g/L SDS pH 7.5). The proteins from the gel were transferred to PVDF membrane using the wet transfer method in transfer buffer (25 mM Tris 119 mM Glycine pH 7.5) at a constant current of 250 mA for 1 h on ice. The PVDF membranes with the transferred proteins were blocked in 20 ml TBST buffer (5 mM Tris containing 0.015 M NaCl and 500 μl/L of Tween 20 pH 7.5) containing 5% milk powder at room temperature for 1 h. The membranes were incubated with primary antibody (typically, 1:1000 dilution) in 1X of TBST buffer containing 5% dry milk overnight at 4 °C. Membranes were washed three times (15 min/wash) with 20 ml of 1X of TBST buffer and incubated with horseradish peroxidase-conjugated secondary antibody (typically, 1:30,000 dilution) in TBST buffer containing 5% milk powder. Following incubation for 1 h at room temperature, the membranes were washed thrice with TBST buffer, overlaid with WestPico, Dura or WestFemto detection reagents (ThermoFisher), and bands were detected using X-ray film.

### Electrophysiology

To quantify the ability of plumbagin and atovaquone to inhibit the NKA, standard whole-cell patch-clamp assay measured the level of the ion current across the membrane. To identify NKA ion currents, inhibitors were used to block Ca^2+^Na/Ca^2+^ and K^+^ channels. The potassium channel currents were inhibited by adding BaCl_2_ to the external solution and removing K^+^ from the internal solution. Ca^2+^ currents were eliminated by the omission of Ca^2+^ from the external solution and adding CdCl_2_ and NiCl_2_, Ca^2+^ channel inhibitors, to the external solution. Na^+^/Ca^2+^ exchange currents were inhibited by removing Ca^2+^ from the internal and external solutions and adding NiCl_2_ to the external solution. Membrane currents in response to the depolarization (up to + 50 mV) and hyperpolarization (up to − 160 mV) were measured. The potential was applied in a stepwise manner from − 160 mV. We applied + 50 mV to induce membrane depolarization and then activating the outward current of the enzyme (3Na^+^ move out of the cell) to maintain the membrane potential. However, applying − 160 mV induced the membrane hyperpolarization and activation of NKA in-ward currents (2 K^+^ go into to the cell). The cell was exposed to the 10 µM of plumbagin or atovaquone, and the inward as well as the outward current was measured using both ramp and voltage steps protocol. The current through the NKA was determined by digitally subtracting the difference between the current before and after the treatment with drugs. The time course of the effect of the drugs on the NKA current at specified voltage pulses was determined. All data were acquired and analyzed using Axopatch 200B amplifier, Digidata, and pClamp-10 Version 10.7 software (Molecular Devices, San Jose, CA). For plotting of electrophysiology data and statistical analysis, we used Excel (Microsoft, Seattle, WA) and Microcal Origin 2019 (OriginLab, Northampton, MA).

### Statistical analysis

The results from all experiments were plotted in GraphPad Prizm software 6.04 for Windows (GraphPad Software La Jolla CA, USA, www.graphpad.com). Statistical analysis was conducted using the features available through this software package. Typically, we used the one way ANOVA test to determine statistical significance and calculate the p-values.

## Supplementary information

Supplementary Information.
