# Supplementary material for: Oxidative stress induced by the anti-cancer agents, plumbagin, and atovaquone, inhibits ion transport through Na+/K+-ATPase
Source: Sci Rep. 2020 Nov 11;10:19585. doi: 10.1038/s41598-020-76342-5 (PMC7659016; doi:10.1038/s41598-020-76342-5)

Oxidative stress induced by the anti-cancer agents, plumbagin, and atovaquone, inhibits ion transport through Na^+/K^+-ATPase

Yousef Alharbi[1,2], Arvinder Kapur[1], Mildred Felder[1], Lisa Barroilhet[1], Bikash R. Pattnaik[3,*], Manish S. Patankar[1,*]

[1]Department of Obstetrics and Gynecology, University of Wisconsin-Madison, Madison WI-53792, USA; yhrby@qu.edu.sa (YA); akaur@wisc.ed (AK); mfelder@wisc.edu (MF); barroilhet@wisc.edu (LB); patankar@wisc.edu (MSP)

[2]Department of Veterinary medicine, Qassim University, Qassim, Saudi Arabia; yhrby@qu.edu.sa (YA)

[3]Department of Pediatrics, Ophthalmology and Visual Sciences, McPherson Eye Research Institute, University of Wisconsin-Madison, Madison WI-53706, USA; pattnaik@wisc.edu (BP)

Supplementary File 1
Whole western blots of Figure 1C

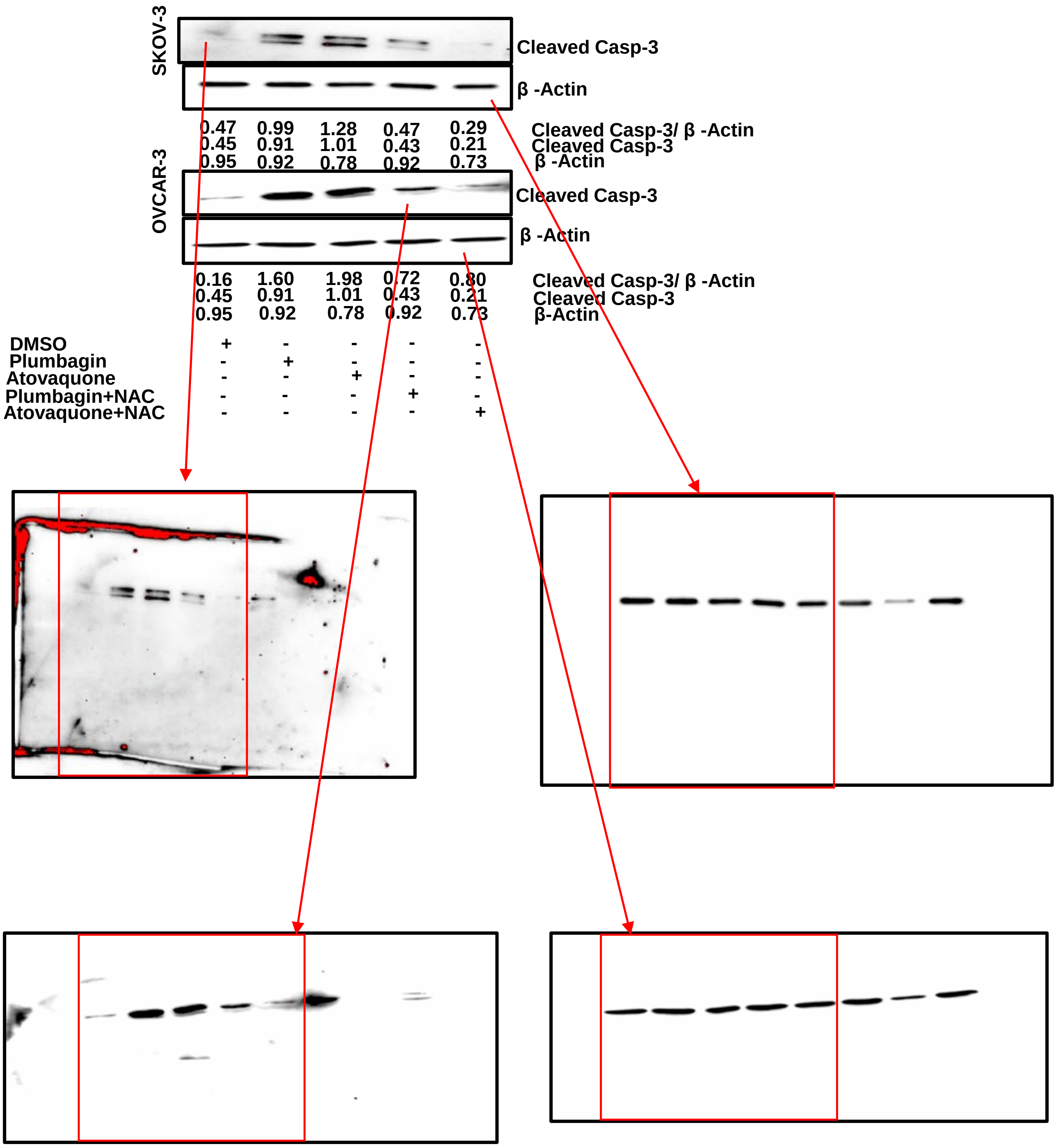

Lanes included in Fig 1C are marked by red box

Supplementary File 2
Whole western blots of Figure 6

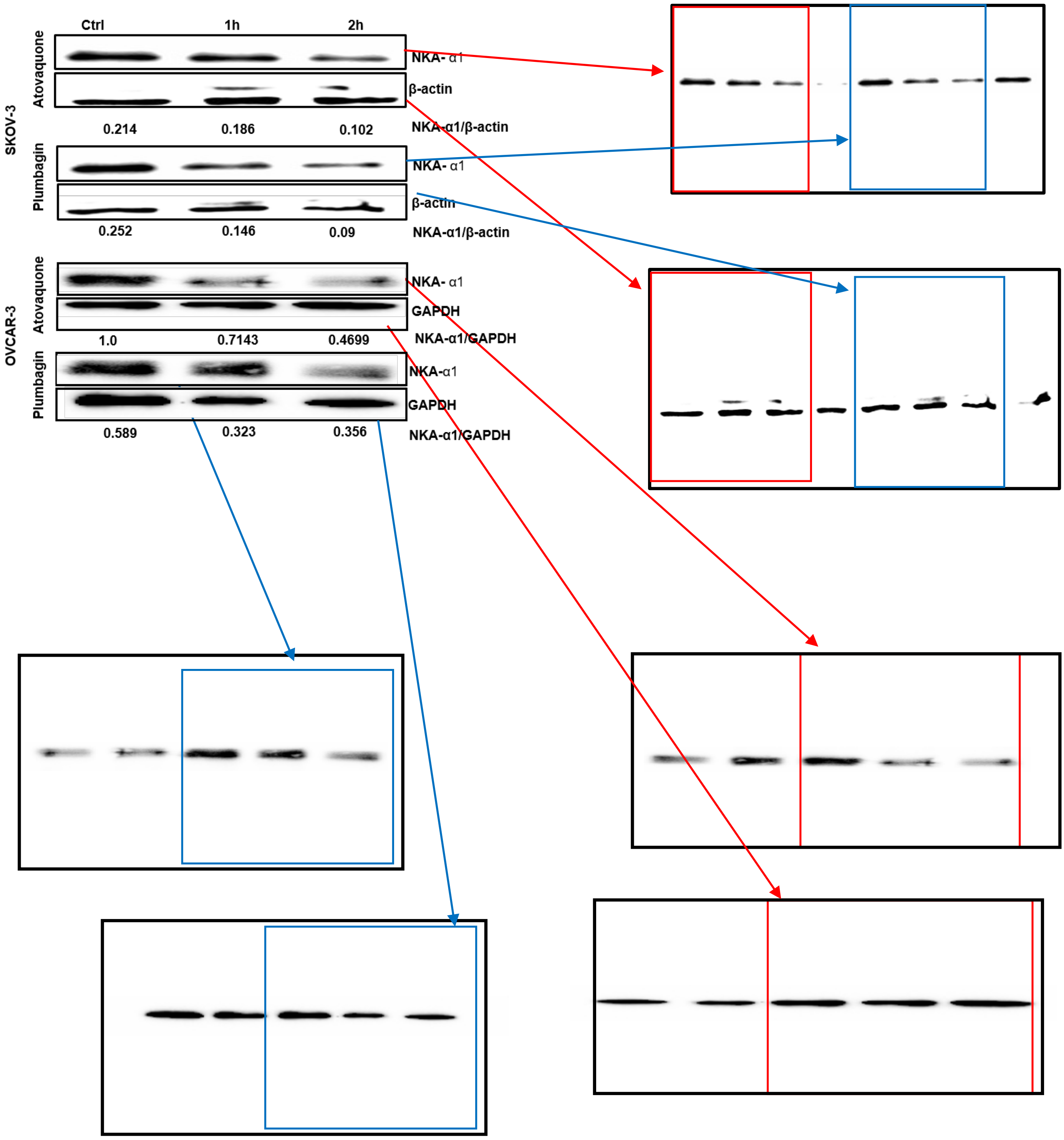

Supplement: Supplementary file 1 — Supplementary Information. [file 41598_2020_76342_MOESM1_ESM.pdf]
